# Evaluating cardiopulmonary resuscitation in *“The Pitt”*: accuracy, clinical realism, and adherence to international resuscitation guidelines

**DOI:** 10.1016/j.resplu.2026.101513

**Published:** 2026-10-07

**Authors:** Siuzanna Kulyk, Anna Carola Hertrich, Fabian Gleibs, Frederic Jaden Aretz, Tim Naumann, Dominic Wichmann, Stefan Kluge, Kevin Roedl

**Affiliations:** Department of Intensive Care Medicine, University Medical Centre Hamburg-Eppendorf, Hamburg, Germany

**Keywords:** Cardiac arrest, In-hospital cardiac arrest, Out-of-hospital cardiac arrest, OHCA, IHCA, TV, Television, *The Pitt*

## Abstract

**Introduction:**

Medical television dramas shape public perceptions of cardiopulmonary resuscitation (CPR). *The Pitt* has been praised for its clinical realism but has not been systematically evaluated. We assessed adherence of televised CPR to current European Resuscitation Council (ERC) and American Heart Association (AHA) guidelines.

**Methods:**

All cardiac arrest (CA) scenarios requiring chest compressions or defibrillation in seasons 1 & 2 (30 episodes) of *The Pitt* were prospectively analysed using a standardized assessment via an electronic case report form based on Utstein-style variables by at least 2 emergency and/or intensive care physicians experienced in CA management.

**Results:**

Seventeen CA were identified. Most occurred in hospital (65%, *n* = 11/17) and had medical causes (59%, *n* = 10/17). ROSC was achieved in 56% (*n* = 9/16), and survival to discharge in 46% (*n* = 6/13). Key elements of the initial assessment, including consciousness and pulse checks, were fully guideline compliant, and correct hand placement was observed in 94% (*n* = 15/16). In contrast, guideline-compliant compression rate were achieved in only 19% (*n* = 3/16) of assessable cases, respectively, and pre-airway 30:2 compression-to-ventilation ratios were absent. Immediate post-shock rhythm reassessments were observed in 78% (*n* = 7/9) of appropriate cases. Nevertheless, no inappropriate shocks were delivered during asystole, and rhythm-based pharmacotherapy was appropriate, although medication timing and sequence could not be reliably assessed.

**Conclusion:**

*The Pitt* provides a highly realistic portrayal of cardiac arrest, although manual CPR technique and some algorithmic details remain imperfect.

## Introduction

Despite substantial advances in resuscitation science, survival following cardiac arrest (CA) remains poor. Survival after out-of-hospital cardiac arrest (OHCA) is approximately 10%, compared with around 25% after in-hospital cardiac arrest (IHCA).^1^ These figures emphasise the importance of early recognition, prompt initiation of high-quality cardiopulmonary resuscitation (CPR), and adherence to evidence-based resuscitation guidelines.^2^, ^3^, ^4^, ^5^

Television and on-demand platforms remain important sources of medical information and contribute to public perceptions of medical emergencies.^6^ Previous studies have demonstrated that medical dramas frequently depict CPR inaccurately, including deviations from guideline-based practice and unrealistically favourable survival outcomes.^7^ Such portrayals may contribute to misconceptions regarding CPR and create unrealistic expectations among viewers.

The medical drama *The Pitt* has gained widespread recognition for its authentic portrayal of emergency medicine. Reaching an estimated audience of more than 10 million viewers in the United States alone, the series may have educational implications and influence public expectations.

The aim of this study was to systematically evaluate the realism of CPR depicted in *The Pitt* by assessing adherence to current European Resuscitation Council (ERC) and American Heart Association (AHA) resuscitation guidelines.^2^, ^3^, ^4^, ^5^

## Methods

### Study design, setting and ethics

We conducted a prospective observational study evaluating the portrayal of CPR in the first 2 seasons of the medical drama *The Pitt*. All 30 episodes (15 per season) were reviewed, and every televised CA scene requiring CPR was screened for inclusion. Resuscitation efforts involving the same patient across multiple scenes or consecutive episodes were considered a single CA event. CPR quality and protocol compliance were evaluated according to contemporary ERC and AHA guidelines^2^, ^3^, ^4^, ^5^ by at least 2 independent physicians. The need for informed consent was waived by the Ethics Committee of the Hamburg Chamber of Physicians because the study involved fictional characters.

### Inclusion criteria

All cardiac arrest scenarios involving chest compressions and/or defibrillation in seasons 1 and 2 were included.

### Data collection

Data were collected using a pre-specified electronic case report form (eCRF). Variables addressed patient demographics, CA circumstances, basic and advanced life support interventions, post-resuscitation care, and clinical outcomes. Variables were selected according to current ERC and AHA algorithms and Utstein-style guidelines.^8^

Only information directly observable or explicitly stated in the episodes was recorded. Variables were classified as “not applicable” when an intervention was irrelevant and as “not assessable” when information was omitted or could not be reliably determined.

All resuscitation scenes were reviewed repeatedly at least three times, with additional viewings when necessary. Following the independent assessments, any uncertainties or discrepancies were discussed among the reviewers and resolved by consensus. Chest compression rate was estimated by counting visible compressions in relation to elapsed video playback time. Compression depth was assessed visually according to observable thoracic displacement because no standardized reference scale was available. These assessments were necessarily subjective because of camera angles, scene cuts, and the absence of calibrated visual references.

Because cinematic editing prevented reliable determination of exact intervention intervals, time-dependent medication dosing was not assessed. Instead, pharmacological management was evaluated according to whether epinephrine and amiodarone were administered when clinically indicated by the presenting rhythm.

### Study definitions

CA was defined as cessation of circulation requiring chest compression and/or defibrillation. Sustained return of spontaneous circulation (ROSC) was defined as stable circulation for at least 20 min. Neurological outcomes were assessed using Cerebral Performance Categories (CPC), with scores 1–2 considered favourable.

### Statistical analysis

Data are presented as absolute numbers and relative frequencies. Percentages were calculated using the number of applicable or assessable cases as the denominator. No inferential statistical analyses were performed because of the descriptive nature of the study. Statistical analysis was conducted using Microsoft Excel. The study was prepared in accordance with STROBE recommendations^9^ (see Supp. Table 1).

## Results

A total of 17 CA events were identified (Table 1). Six-Teen scenes depicted patient resuscitations and 1 represented a CPR training scenario. Most CAs occurred in the emergency department (*n* = 11/17, 65%), involved adults (*n* = 15/17, 88%), and had a primary medical cause (*n* = 10/17, 59%). CA was witnessed in 75% of applicable cases (*n* = 9/12). An initial shockable rhythm was documented in 53% (*n* = 9/17), while 18% (*n* = 3/17) were non-shockable and 30% (*n* = 5/17) were not available.

**Table 1:** Baseline characteristics, arrest circumstances, and clinical outcomes of cardiac arrest events in *The Pitt.*

**Characteristic**		***n*/*N* (%)**
Total CA events		17 (100)
Age	Adult patients (≥18 years)	15/17 (88)
	Paediatric patients (<18 years)	1/17 (6)
	CPR training scenario	1/17 (6)
Sex	Male sex	10/17 (59)
	Female sex	6/17 (35)
	Resuscitation training manikin	1/17 (6)
Location of CA	OHCA	5/17 (29)
	IHCA	11/17 (65)
	CA location not applicable	1/17 (6)
Aetiology of CA	Medical aetiology	10/17 (59)
	Traumatic aetiology	6/17 (35)
	CPR training scenario	1/17 (6)
Witnessed CA		9/12 (75)
Initial rhythm	Shockable rhythm	9/17 (53)
	Non-shockable rhythm	3/17 (18)
	Rhythm not available	5/17 (30)
ROSC		9/16 (56)
Outcome	Survival to hospital discharge	6/13 (46)
	Good neurological outcome reported^a^	3/6 (50)
	Good quality of life after CPR reported^a^	3/6 (50)

*Abbreviations:* CA – cardiac arrest; CPR – cardiopulmonary resuscitation; IHCA – in-hospital cardiac arrest; OHCA – out-of-hospital cardiac arrest; ROSC – return of spontaneous circulation.

a Data reflect only cases with explicitly documented post-resuscitation status; the absence of follow-up data for the remaining cases does not imply a poor functional or neurological outcome.

Among actual patient encounters, ROSC was achieved in 9 out of 16 patients (56%). Survival to hospital discharge was documented in 46% of patients with available outcome data (*n* = 6/13). A favourable neurological status and good post-arrest quality of life were each explicitly reported in 50% of these cases (*n* = 3/6) (Table 1).

During initial assessment, consciousness checks, pulse checks, and assessment duration under 10 s achieved 100% guideline compliance in all visualized instances. Chest compressions were initiated promptly, and correct hand positioning was maintained in 94% (*n* = 15/16) of applicable events (Fig. 1).

**Fig. 1:**
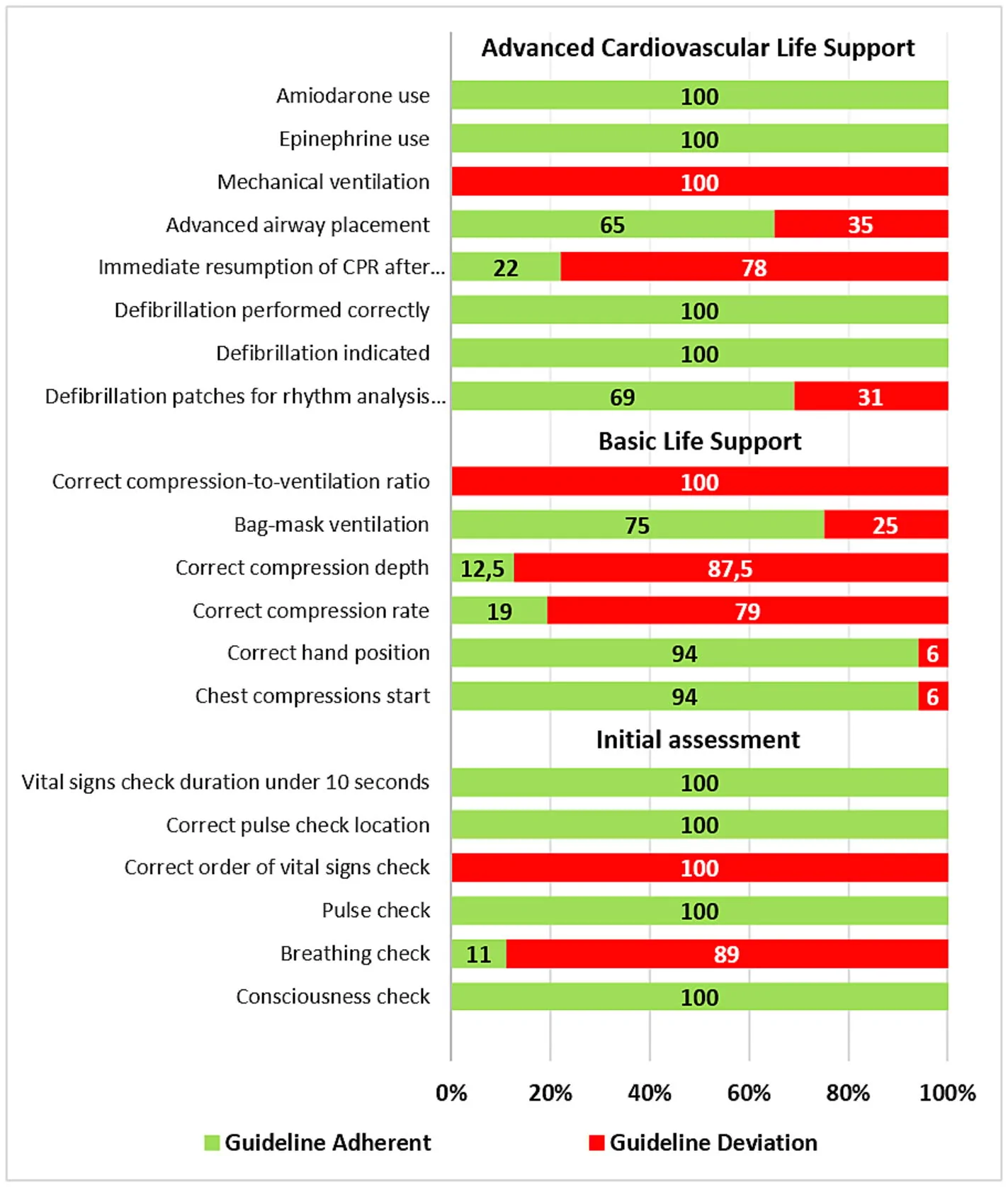
**Guideline adherence and procedural deviations during televised cardiopulmonary resuscitation in *The Pitt***.

In contrast, technical CPR quality was substantially compromised. Correct compression rate was achieved in only 19% (*n* = 3/16) of assessable scenarios. None of the evaluated events maintained the standard 30:2 compression-to-ventilation ratio before advanced airway placement. Advanced airway placement was shown in 65% (*n* = 11/17), but guideline-conforming mechanical ventilation thereafter was not observed (Fig. 1).

In 78% (*n* = 7/9) of shock delivery scenarios, immediate rhythm reassessment occurred after defibrillation instead of uninterrupted resumption of chest compressions, representing a significant algorithmic deviation (Fig. 1).

## Discussion

To our knowledge, this is the first study to systematically evaluate CPR quality in The Pitt, a television series widely regarded as an authentic portrayal of emergency medicine. Overall, the series demonstrated a high degree of clinical realism, although relevant deviations from contemporary ERC and AHA guidelines were identified.

Several deviations, particularly those involving compression depth and manual CPR technique, may reflect production constraints and actor safety rather than a lack of medical understanding. Importantly, the series also portrayed key evidence-based principles, including appropriate withholding of defibrillation in asystole. These findings suggest that medical accuracy was actively considered during production despite the practical limitations of filming resuscitation scenes.

Compared with previously evaluated medical dramas,^6^, ^10^, ^11^, ^12^, ^13^ *The Pitt* demonstrated substantial alignment with clinical epidemiology. Medical causes accounted for 59% of cases, contrasting with the predominance of traumatic aetiologies reported in several earlier television studies. ROSC occurred in 56% of patient encounters, and post-resuscitation deaths and prolonged resuscitation attempts were depicted, contributing to a more realistic portrayal of CA outcomes (Table 2).

**Table 2:** Systematic overview of demographic parameters, guideline adherence, and survival outcomes across CPR television studies and clinical registries.

Parameter		Diem et al. (1996) (1)	Harris and Willoughby (2009) (2)	Hinkelbein et al. (2014) (3)	Hörburger et al. (2014) (4)	Portanova et al. (2015) (5)	Present Study	Real-world data			
								**Europa**		**USA**	
								**OHCA (6, 7)**	**IHCA (6, 8)**	**OHCA (9)**	**IHCA (10)**
TV series		*ER* *Chicago Hope* *Rescue 911*	*Casualty* *Holby City* *Grey's Anatomy* *ER*	*ER*	*Star Trek*	*Grey’s Anatomy* *House M.D.*	*The Pitt*^a^	NA	NA	NA	NA
Number of episodes, *n*		97	88	174	526	91	30	NA	NA	NA	NA
Number of CPR-scenes, *n*		60	70	136	96	46	17	25,171^b^	NA, incidence of 1.5–2.8 per 1000 admissions	70,027^b^	235,560^b^
Age	Paediatric patients (<18 years), *n* (%)	26 (43%)	NA, average age and age range of patients is 36.34 years and 8 months–85 years	NA, average age and 25/75 percentile are 28 and 15/40 years	excluded	8 (22%)	1 (6%)	NA, average age and age range are 67.6 years and 0–107 years	excluded	NA, mean age and standard deviation were 64.1 years and 18.2 years)	excluded
	Adult patients (≥18 years), *n* (%)	34 (57%)			96 (100%)	29 (78%)	15 (88%)		100%		235,560 (100%)
Sex	Male, *n* (%)	44 (73%)	48 (69%)	90 (66%)	73 (76%)	24 (65%)	10 (59%)	16,462 (65%)	NA (ca. 64%)	42,918 (61%)	NA
	Female, *n* (%)	16 (27%)	22 (32%)	46 (34%)	23 (24%)	13 (35%)	6 (35%)	8079 (35%)	NA (ca. 36%)	27,109 (39%)	NA
Location of CA	OHCA, *n* (%)	36 (60%)	NA	NA	42 (44%)*	5 (11%)	5 (29%)	25,171 (100%)	0%	100%	0%
	IHCA, *n* (%)	24 (40%)			54 (56%)*	41 (89%)	11 (65%)	0 (0%)	100%	0%	235,560 (100%)
Aetiology of CA	Traumatic aetiology, *n* (%)	21 (35%)	30 (43%)	77 (57%)	64 (67%)	18 (39%)	6 (35%)	982 (4%)	uncommon	NA	NA
	Medical aetiology, *n* (%)	25 (42%)	28 (40%)	12 (9%)	15 (16%)	13 (28%)	10 (59%)	22,931 (91%)	majority	NA	NA
Witnessed CA, *n* (%)		NA	NA	109 (80%)	91 (95%)	NA	9 (53%)	15,824 (67%)	NA	33,075 (47%)	205,398 (87%)
Initial rhythm	Shockable rhythm, *n* (%)	NA	NA	NA	NA	NA	9 (53%)	4792 (20%)	NA	16,229 (23%)	37,465 (16%)
	Non-shockable rhythm, *n* (%)	NA	NA	NA	NA	NA	3 (18%)	18,958 (75%)	NA	53,784 (77%)	198,095 (84%)
ROSC, *n* (%)		46 (77%)	32 (46%)	52 (38%)	11 (12%)	32 (70%)	9 (53%)	8189 (33%)	NA (ca. 58%)	NA	165,831 (71%)
Outcome	Survival to hospital discharge, *n* (%)	22 (37%)	NA	7 (5%)	NA	23 (50%)	6 (35%)	1809 (8%)	NA (ca. 21%)	NA (ca. 7.5%)	51,823 (22%)
	Good neurological outcome, *n* (%)	NA	NA	NA	10 (10%)	NA	3 (18%)	NA	NA	NA	34,392 (15%)
Common mistakes in CPR	Incorrect hand position, *n* (%)	NA	NA	61 (45%)	NA	NA	1 (6%)	NA	NA	NA	NA
	Incorrect compression rate, *n* (%)	NA	NA	88 (65%)	NA	NA	13 (76%)	NA	NA	NA	NA
	Incorrect compression depth, *n* (%)	NA	NA	125 (92%)	NA	NA	NA	NA	NA	NA	NA
	Incorrect compression-to-ventilation ratio, *n* (%)	NA	NA	NA	NA	NA	16 (94%)	NA	NA	NA	NA
	Not indicated Defibrillation, *n* (%)	NA	0 (0%)	NA, in many scenes	NA	NA	0 (0%)	NA	NA	NA	NA
	Incorrect Epinephrine use, *n* (%)	NA	NA	67 (49%)	NA	NA	0 (0%)	NA	NA	NA	NA
	Incorrect Amiodarone use, *n* (%)	NA	NA	103 (76%)	NA	NA	0 (0%)	NA	NA	NA	NA

*Abbreviations:* TV – television; ER – Emergency Room; CPR – cardiopulmonary resuscitation; CA – cardiac arrest; IHCA – in-hospital cardiac arrest; OHCA – out-of-hospital cardiac arrest; ROSC – return of spontaneous circulation; EuReCa – European Registry of Cardiac Arrest; GRR – German Resuscitation Registry; AHA – American Heart Association; GWTG-R – Get with The Guidelines®–Resuscitation; NA – not available.

* Because the spacecraft represents the primary healthcare environment within the fictional universe of the series, it was considered functionally equivalent to a hospital for study purposes. Therefore, cardiac arrests occurring inside the spacecraft were classified as in-hospital cardiac arrests (IHCA), while those occurring outside the spacecraft were classified as out-of-hospital cardiac arrests (OHCA). This pragmatic classification was chosen to allow comparison with established Utstein definitions and previously published studies evaluating CPR in television dramas.

a The percentage was calculated based on the total number of resuscitation scenarios, which amounted to 17, including cases where the information was ‘not applicable’ or ‘not assessable’.

b This refers to actual cases of CPR of patients, according to the registry’s data.

Nevertheless, shockable rhythms were substantially overrepresented compared with contemporary European and North American CA registries^14^, ^15^, ^16^, ^17^, ^18^ (Table 2). This may reflect the greater dramatic potential of ventricular fibrillation and pulseless ventricular tachycardia, which allow the depiction of defibrillation and advanced life support interventions.

The observed deviations in CPR technique are clinically relevant. High-quality chest compressions are fundamental to successful resuscitation, and inadequate compression rate or depth may reduce coronary and cerebral perfusion. Similarly, interruptions in compressions and deviations from recommended ventilation strategies may adversely affect outcomes. While some technical inaccuracies, particularly compression depth, may be unavoidable because of actor safety, other deviations could potentially be corrected without compromising dramatic storytelling.

Medical dramas are increasingly recognised as sources of health-related information for the general public.^19^ Viewers may perceive medically realistic series as credible representations of clinical practice.^20^ Consequently, inaccurate CPR depictions may reinforce misconceptions regarding evidence-based resuscitation. Conversely, accurate portrayals may support public understanding of CPR and resuscitation practice.

These findings also highlight an opportunity for closer collaboration between television producers and resuscitation experts. Many identified inaccuracies could potentially be addressed through relatively minor adaptations of scripts or choreography without compromising narrative quality.

### Limitations of the study

This study has several limitations. First, the analysis was based on a fictional television series rather than real-life resuscitation events. Directorial decisions, camera angles, editing, and time constraints may limit assessment of certain measures. Second, some CPR components were not explicitly shown and therefore could not be assessed. Third, rapid scene changes made it difficult to determine the duration of individual CPR periods, preventing reliable assessment of medication timing. Fourth, only observable actions could be evaluated, meaning that interventions performed off-screen could not be assessed.

Finally, CPR technique is inevitably influenced by actor safety. Performing guideline-recommended compression depth and force on healthy actors is neither feasible nor ethically acceptable. Deviations in compression depth and technique may therefore partly reflect practical production constraints rather than a lack of medical accuracy.

## Conclusion

*The Pitt* demonstrates a high degree of overall clinical realism despite substantial deviations from guideline-recommended CPR technique and selected resuscitation algorithms. Medication use according to presenting rhythm was appropriate, although timing and sequence could not be reliably assessed. The recurrent immediate rhythm reassessment following defibrillation represents a notable deviation and may reflect a systematic production or scripting issue. Greater alignment with current ERC and AHA guidelines could further enhance the series’ educational value and support a more accurate public understanding of evidence-based resuscitation.

## CRediT authorship contribution statement

**Siuzanna Kulyk:** Writing – original draft, Methodology, Formal analysis, Conceptualization. **Anna Carola Hertrich:** Writing – review & editing, Investigation, Data curation. **Fabian Gleibs:** Writing – review & editing, Formal analysis, Data curation. **Frederic Jaden Aretz:** Investigation, Data curation. **Tim Naumann:** Writing – review & editing, Investigation. **Dominic Wichmann:** Writing – review & editing, Supervision, Project administration, Investigation, Formal analysis. **Stefan Kluge:** Writing – review & editing, Investigation, Formal analysis, Data curation. **Kevin Roedl:** Writing – review & editing, Writing – original draft, Validation, Supervision, Project administration, Methodology, Investigation, Formal analysis, Data curation, Conceptualization.

## Ethics approval

This study protocol was reviewed and approved by the Ethics Committee of the Hamburg Chamber of Physicians in Hamburg, Germany (“Ethikkomission der Ärztekammer Hamburg”), approval No.: 2026–300771-WF.

## Consent for publication

Not applicable.

## Availability of data and materials

The datasets supporting the conclusions of this article are included within the article. Further enquiries can be directed to the corresponding author.

## Declaration of Generative AI and AI-assisted technologies in the writing process

The authors used ChatGPT (OpenAI, San Francisco, CA, USA) exclusively for language editing and improvement of English grammar and clarity. All scientific content, analysis, interpretation, conclusions and references were developed by the authors. The authors reviewed and edited all outputs and take full responsibility for the content of this manuscript.

## Funding sources

This study was supported exclusively by institutional funds of the Department of Intensive Care Medicine of the University Medical Center Hamburg-Eppendorf.

## Declaration of competing interest

SiKu, ACH, FG, FA and KR do not report any conflicts of interest. DW received consulting honoraria from Biosergen, Gilead, Mundipharma, Shionogi and Solventum; lecture honoraria from 3M, Astra Zeneca, Eumedica, Gilead, InfectoPharm, Mundipharma, MSD, Pfizer, Shionogi and Solventum; research support from F2G and Shionogi and is holding stocks from Bayer. SK received research support from Biotest, CytoSorbents, Daiichi Sankyo, Fresenius Medical Care; lecture fees from ADVITOS, Biomerieux GmbH, CSL Behring, Gilead, MSD, Pfizer, Shionogi; consultant fees from ADVITOS, Gilead and Pfizer.

## Appendix A Supplementary material

Supplementary Table 1 STROBE checklist for the observational study evaluating CPR depictions in *The Pitt*. STROBE Statement.
